# Establishment and validation of exhausted CD8+ T cell feature as a prognostic model of HCC

**DOI:** 10.3389/fimmu.2023.1166052

**Published:** 2023-04-03

**Authors:** Jihang Shi, Guangya Li, Lulu Liu, Xiandun Yuan, Yafei Wang, Ming Gong, Chonghui Li, Xinlan Ge, Shichun Lu

**Affiliations:** ^1^ Medical School of Chinese People’s Liberation Army (PLA), Beijing, China; ^2^ Faculty of Hepato-Pancreato-Biliary Surgery, Chinese People’s Liberation Army (PLA) General Hospital, Beijing, China; ^3^ Institute of Hepatobiliary Surgery of Chinese PLA, Beijing, China; ^4^ Ministry of Education (MOE) Key Laboratory of Cell Proliferation and Differentiation, College of Life Sciences, Peking-Tsinghua Center for Life Sciences, Peking University, Beijing, China; ^5^ Peking University-Tsinghua University-National Institute of Biological Science Joint Graduate Program, College of Life Science, Peking University, Beijing, China; ^6^ Department of Rheumatology and Immunology, Peking University Third Hospital, Beijing, China

**Keywords:** CD8+ T, T cell exhaustion, hepatocellular carcinoma, prognostic model, survival analysis

## Abstract

**Objectives:**

The exhausted CD8+T (Tex) cells are a unique cell population of activated T cells that emerges in response to persistent viral infection or tumor antigens. Tex cells showed the characteristics of aging cells, including weakened self-renewal ability, effector function inhibition, sustained high expression of inhibitory receptors including PD-1, TIGIT, TIM-3, and LAG-3, and always accompanied by metabolic and epigenetic reprogramming. Tex cells are getting more and more attention in researching immune-related diseases and tumor immunotherapy. However, studies on Tex-related models for tumor prognosis are still lacking. We hope to establish a risk model based on Tex-related genes for HCC prognosis.

**Methods:**

Tex-related GEO datasets from different pathologic factors (chronic HBV, chronic HCV, and telomere shortening) were analyzed respectively to acquire differentially expressed genes (DEGs) by the ‘limma’ package of R. Genes with at least one intersection were incorporated into Tex-related gene set. GO, KEGG, and GSEA enrichment analyses were produced. Hub genes and the PPI network were established and visualized by the STRING website and Cytoscape software. Transcription factors and targeting small molecules were predicted by the TRUST and CLUE websites. The Tex-related HCC prognostic model was built by Cox regression and verified based on different datasets. Tumor immune dysfunction and exclusion (TIDE) and SubMap algorithms tested immunotherapy sensitivity. Finally, qRT-PCR and Flow Cytometry was used to confirm the bioinformatic results.

**Results:**

Hub genes such as AKT1, CDC6, TNF and their upstream transcription factor ILF3, Regulatory factor X-associated protein, STAT3, JUN, and RELA/NFKB1 were identified as potential motivators for Tex. Tex-related genes SLC16A11, CACYBP, HSF2, and ATG10 built the HCC prognostic model and helped with Immunotherapy sensitivity prediction.

**Conclusion:**

Our study demonstrated that Tex-related genes might provide accurate prediction for HCC patients in clinical decision-making, prognostic assessment, and immunotherapy. In addition, targeting the hub genes or transcription factors may help to reverse T cell function and enhance the effect of tumor immunotherapy.

## 1 Introduction

Hepatocellular carcinoma (HCC), accounting for more than 90% of primary liver tumors, is now the world’s fifth most common cause of cancer. The five-year survival rate for liver cancer was 18% and second only to that for pancreatic cancer (1). Important risk factors for hepatocellular carcinoma include hepatitis liver disease (hepatitis B and C), alcoholic liver disease, and nonalcoholic fatty liver disease (2). Due to its insidious progression, most patients were diagnosed at advanced stages. RFA, TACE, TKI, and immunotherapy are widely used in advanced liver cancer (3). Advanced therapies, including gene therapy and immunotherapy, have shown incredible effects, and more and more combination therapies are also being carried out in clinical studies, offering hope for the treatment of patients with advanced liver cancer (4–6).

Nevertheless, the role of immune cells, especially exhausted T cells, in developing and progressing HCC at different stages and whether exhaustion features can be utilized as a diagnostic and prognostic marker for HCC remains unclear. T cell exhaustion, usually called exhausted CD8+T (Tex) cells, is a hypofunctional state of T lymphocytes, endowing them to lose their ability to eliminate cancer cells effectively (7, 8). Prolonged antigen exposure and persistent inflammatory stimuli are thought to be potential mechanisms that drive T cells to an exhaustion state (9, 10). Advanced HCC is often accompanied by an immune exhaustion station, characterized by the accumulation of PD-1^hi^ CD8+T cells and exhaustion of tumor-antigen-specific CD8+T cells (11). T-cell exhaustion was associated with overall survival in HCC patients of different ethnicities and etiologies (12, 13). In HCC, the T cell exhaustion factor is particularly important for several reasons. On the one hand, due to its unique physiological structure, the liver plays an important role in promoting immune tolerance (14). And the immune tolerance microenvironment contributes to the latent growth of malignant hepatocytes. Research demonstrates that the numbers of Tregs, MDSCs, and exhausted T cells were increased in patients with advanced liver cancer compared with normal controls (15, 16). On the other hand, chronic HBV and HCV infection are important etiologies of HCC, leading to liver fibrosis and chronic inflammatory reaction (17). All these factors aggravate the development of Tex.

In conclusion, Tex, a reliable immunophenotype of HCC, may be related to the efficacy of immunotherapy and prognosis (18). However, there is no Tex-based prognostic model to systematically evaluate Tex-related genes and predict the overall prognosis of HCC patients. Therefore, establishing a Tex-based prognostic model for diagnosing and treating HCC has important clinical significance.

In the first step of this study, we analyzed data sets of exhausted CD8+T (Tex) cells from different etiologies (HBV, HCV, TOLER) to find Tex-related co-differentially expressed genes and analyzed the hub gene networks and associated signaling pathways. Then we constructed a Tex-related prognostic model based on the TCGA database and validated it in an independent ICGC(International Cancer Genome Consortium) database. The results demonstrated that the Tex-related genes are related to HCC prognosis, and the Tex-related prognostic model would be effective in clinical decision-making, prognostic assessment, and immunotherapy for HCC treatment.

## 2 Research methods

### 2.1 Datasets collection

This article’s related datasets included HBV, HCV, and Telomere shortening data of exhausted CD8+ T cells from the Gene Expression Omnibus (GEO) database. The HBV dataset (GSE67801) contains four HBV-induced Tex samples and five control samples. The HCV dataset (GSE111449) contains seven HCV-infected Tex samples and five healthy samples, and the telomere-shortening dataset (GSE77525) has four telomere-shortening CD8+T cell samples and four healthy samples. TCGA-LIHC cohort containing RNA sequencing and corresponding clinical information of 369 HCC samples were downloaded from the TCGA website until January 1, 2023. ICGC (LIRI-JP) cohort comprises 231 HCC samples from the ICGC portal.

### 2.2 Identification of common DEGs between HBV, HCV, and telomere shortening

DEGs of three GEO, TCGA, and ICGC datasets were analyzed with standard | Log2Fold Change | > 1 and | adj. P value | < 0.05. The ‘limma’ package in RStudio software (version 4.2.2) was used. Common DEGs were produced at least one intersection by the ‘Venn’ website (http://bioinformatics.psb.ugent.be/webtools/Venn/) for Tex gene list construction.

### 2.3 Pathway enrichment analysis of common DEGs

The ‘clusterProfiler’ package was used to process GO and KEGG enrichment analysis in R software (version 4.2.2). Other packages, including ‘dplyr,’ ‘org.Hs.eg.db,’ ‘circlize,’ ‘RColorBrewer,’ ‘ggplot2,’ ‘enrichplot,’ ‘ggpubr,’ and ‘ComplexHeatmap’ were used for data Annotation and Visualization. P-value < 0.05 was set as the cutoff criterion for common DEGs.

### 2.4 PPI network analysis based on common DEGs

PPI networks were established by Search Tool for the Retrieval of Interacting Genes Database (STRING) based on the combined score > 0.9 and visualized by Cytoscape 3.9.1 (version 4.2.2) to reveal the interactions among proteins of common DEGs.

### 2.5 Establishment and validation of the Tex-related prognostic model

During the model construction, cases with OS time less than 90 days in TCGA and ICGC datasets were excluded. The TCGA data were first randomly divided into the training and testing sets utilizing package “caret” in R. Univariate Cox proportional hazard regression analysis by R package “survival” was used to screen prognostic-related Tex DEGs in the training group (p < 0.05). The R package “glmnet” was utilized for Lasso regression. Then, the multivariable Cox proportional risk regression analysis was carried out to establish the prognosis model of HCC based on Tex-related genes in TCGA. The risk score formula is as follows: $\sum_{i=A}^{N}\left(coefi\times iexpression\right)$ , where coef means regression coefficient. The HCC patients in the training, testing, and ICGC validation sets were separated into high- and low-risk groups based on the median risk score. The survival analysis and the receiver operator characteristic (ROC) curves between the two risk groups were carried out using the “survminer,” “survival,” and “timeROC” R packages.

### 2.6 Immunotherapy reactivity

Tex-related HCC risk score was used to predict the patients’ responsiveness to immune checkpoint blocks (ICB). IMvigor210 cohort (http://research-pub.gene.com/IMvigor210CoreBiologies/) containing transcriptomic and corresponding clinical data of HCC patients receiving anti-PD-L1 agent (atezolizumab) treatment. The GSE78220 cohort comprises transcriptomic data from melanomas patients receiving anti-PD-1 checkpoint inhibition therapy.

### 2.7 Independence factors of HCC

Univariate Cox and multivariate Cox regression analyses were utilized to evaluate if the risk score and other clinical characteristics were independent variable factors for HCC.

### 2.8 The stimulation and expansion of T cells

The blood samples were collected from healthy donors (Chinese PLA General Hospital, 2017YFA003003). Then the peripheral blood mononuclear cells (PBMCs) were isolated using Ficoll density gradient centrifugation. The isolated human PBMCs were cultured in complete culture media, including advanced RPMI 1640 media (Gibco, 12633012), supplemented with 10% human serum (Sigma, NIST909C), 200U/ml IL2 (MCE, HY-P7039) and 1% penicillin/streptomycin (Pen/Strep, 15140163), at 37°C and 5% CO_2_ concentration. MagniSort Human T cell Enrichment Kit (Invitrogen, 8804-6810-74) was used to obtain CD3+ cells. The complete culture media were mixed with magnetic beads coated with anti-CD3/CD28 mAbs (Invitrogen, 11141D) to stimulate and expand the CD3+ T cells. T cells were mixed with magnetic beads at a ratio of 1:1 beads-to-cells. During each induction cycle, 2.5 × 10^6^ cells in a 5 mL/well volume were plated in a 6−well culture plate at 37°C/5% CO_2_ for four days. Then magnetic beads were washed away, and cells were cultured in a culture medium for two days. Flow analysis of the T cell exhaustion state was performed after each cycle.

### 2.9 Quantitative real-time polymerase chain reaction

After removing beads from the CD3+ T cells by magnet or grinding liver tissue, total RNA was isolated using the Direct-zol RNA Kits (ZYMO Research, R2052). Then RNA was converted to cDNA using the TransScript First-Strand cDNA Synthesis SuperMix (TransGen Biotech, AT311). qRT-PCR analysis was performed using the KAPA SYBR^®^ FAST qPCR Kit (KAPA Biosystems, KK4601) on a Bio-Rad CFX384TM Real-time System. The quantified values were normalized by housekeeping genes (β-ACTIN or RRN18S). The qRT-PCR primer sequences were provided in **Table 1**, and the data were analyzed using the ΔΔCt method.

**Table 1:** Primer sequences for real-time PCR in the study.

Gene	Forward primer (5’ to 3’)	Reverse primer (5’ to 3’)
18S	GTAACCCGTTGAACCCCATT	CCATCCAATCGGTAGTAGCG
β-actin	CTCCATCCTGGCCTCGCTGT	ACTAAGTCATAGTCCGCCTAGA
AKT1	AGCGACGTGGCTATTGTGAAG	GCCATCATTCTTGAGGAGGAAGT
CDC6	CCAGGCACAGGCTACAATCAG	AACAGGTTACGGTTTGGACATT
ILF3	AGCATTCTTCCGTTTATCCAACA	GCTCGTCTATCCAGTCGGAC
SLC16A11	CGTGGAGGCTTCTCTCACAG	CGTAGGACAGCCCGTTTATCG
CACYBP	CTCCCATTACAACGGGCTATAC	GAACTGCCTTCCACAGAGATG
HSF2	AGAATGAGTCCCTTTGGAAGGA	TTCTTTTGGGCTCCATTAGTGTT
ATG10	AGACCATCAAAGGACTGTTCTGA	GGGTAGATGCTCCTAGATGTGAC

### 2.10 Flow cytometry and apoptosis assays

After 1×10^6^ cells were fixated and permeabilizated, resuspended with 200 µl 1×Perm/Wash Buffer and stained with antibodies APC anti-PD1 (Biolegend, 329908), FITC Anti-Tim3 (Biolegend, 345022), and then detected on the Flow Cytometer.

### 2.11 Other methods and statistical analysis

GeneMANIA (http://www.genemania.org) was used to find genes related to Tex hub genes. The tool was used to visualize the gene networks, construct the protein-protein interaction (PPI) network, and generate hypotheses about gene function. Transcription regulators upstream of hub genes were searched in the TRRUST database (https://www.grnpedia.org/trrust/) with the FDR < 0.05. Small molecules reported to target these transcription factors were obtained by the Clue website (https://clue.io/). All bioinformatics statistical analyses were performed by R (version 4.2.2) and RStudio. p <0 .05 was considered statistically significant. Statistical flow cytometry and qRT-PCR data tests were performed using GraphPad Prism software. Student t-test (two-tailed) was used for comparisons between two independent conditions. p <0 .05 was considered statistically significant.

## 3 Results

### 3.1 Identification of Tex-related genes

**Figure 1:**
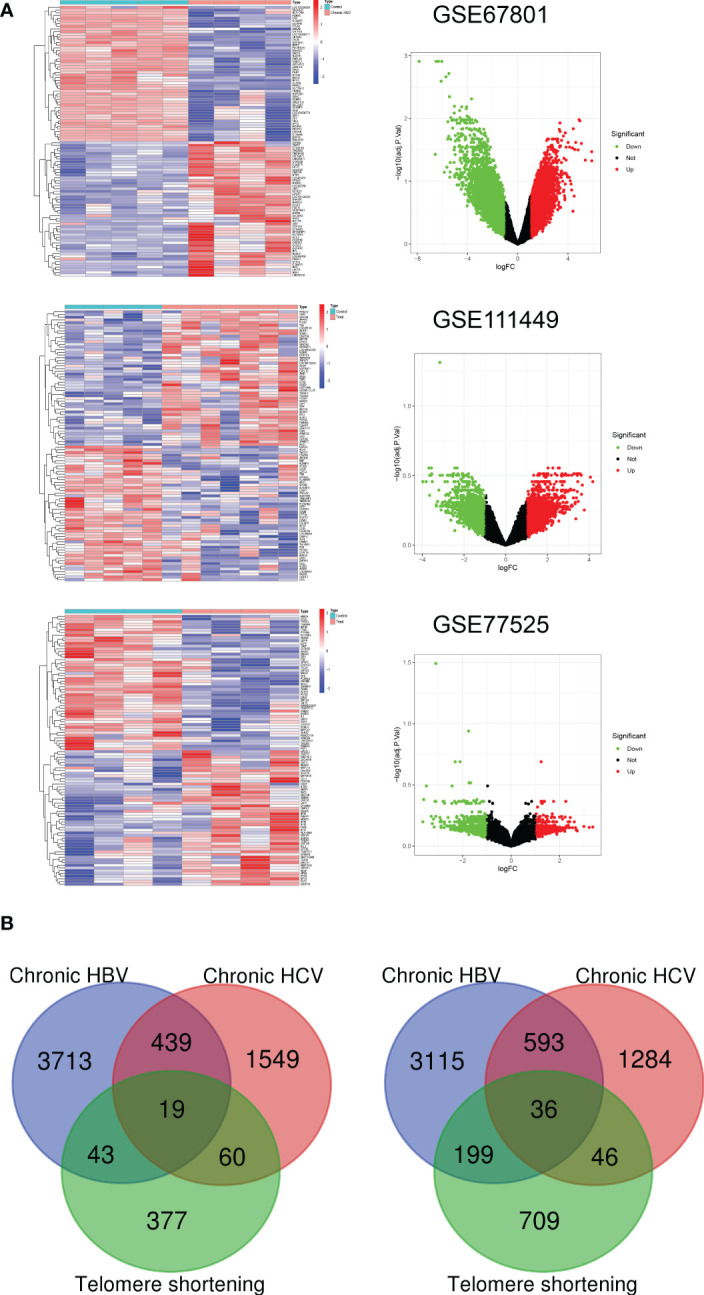
Construction of Tex-related gene list. **(A)** Heatmap and Volcano plot of the DEGs between Tex cells and CD8 T control cells from three GEO datasets. Green represents downregulated DEGs, red represents upregulated DEGs, and black represents no difference. **(B)** Venn diagram demonstrates the co-upregulated and co-downregulated DEGs for the Tex-related gene list.

GEO datasets (GSE67801, GSE111449, and GSE77525) containing three etiologies (chronic HBV, HCV infection, and telomere shortening) induced exhausted CD8+T (Tex) cells and their normal control data were selected to screen for Tex genes (**Figure 1A**). Up-regulated DEGs and down-regulated-DEGs were analyzed with standard | Log2Fold Change | > 1 and | adj. P value | < 0.05 followed by intersection operation. The VENN diagram demonstrated that 561 co-upregulated genes and 834 co-downregulated genes constituted 1435 Tex-related gene lists (**Figure 1B**, **Supplementary 1**).

### 3.2 PPI network construction and hub gene selection

**Figure 2:**
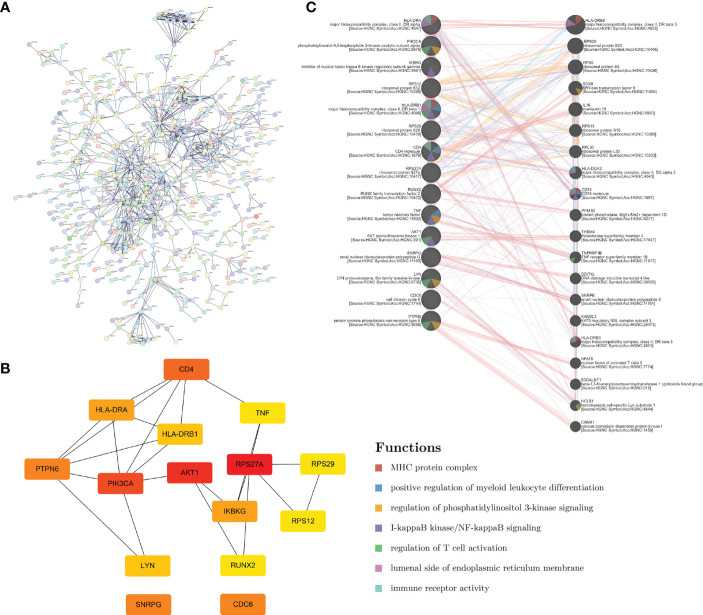
Hub genes extraction and PPI network construction. **(A)** PPI network of Tex-related genes constructed by STRING. **(B)** The top 15 hub genes and their relationship were calculated and visualized by Cytoscape. **(C)** GeneMANIA database helps predict the hub gene interaction network based on gene correlations such as colocalization, coexpression, and shared protein domains. The 15 hub genes were listed on the left side, and the top 20 mostly related neighboring genes are shown on the right side.

By using the STRING database, we built a PPI network containing 1021 edges and 526 nodes to explore further the correlation between genes in the Tex gene list (**Figure 2A**). Then, CytoHubba in Cytoscape was used to identify the 15 hub genes based on degree method scores in the PPI network, including TNF, AKT1, RPS29, RPS27A, PTPN6, RUNX2, CD4, LYN, HLA-DRB1, IKBKG, CDC6, HLA-DRA, SNRPG, RPS12, and PIK3CA (**Figure 2B**). Hub genes were analyzed further by GeneMANIA to explore their interacting genes and predict their correlations such as colocalization, coexpression, and shared protein domains. The hub genes are on the left side, while the interacting genes are on the right. Function analysis showed that these genes were enriched in the MHC protein complex, positive regulation of myeloid leukocyte differentiation, PI3K, and NF-κB signaling (**Figure 2C**).

### 3.3 Functional enrichment analysis of Tex-related hub genes

**Figure 3:**
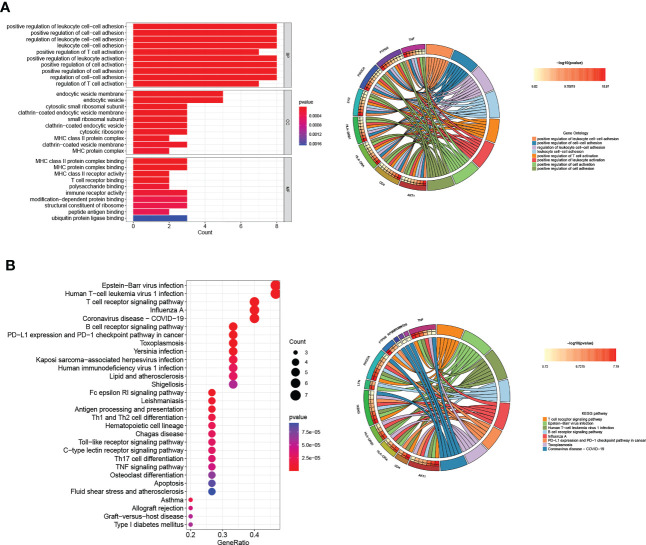
GO and KEGG pathway analyses of Hub genes. **(A)** The top ten GO functional enrichment analyses of the hub genes in biological process (BP), cellular component (CC), and molecular function (MF) groups, respectively. **(B)** The top 30 significant KEGG signal pathways of the hub genes.

We used a ‘cluster profiler’ package in R software to analyze these Tex-related hub genes’ biological role and function to perform GO and KEGG enrichment analyses. GO enrichment analysis included three categories: biological process (BP), cell composition (CC), and molecular function (MF). Regarding BP, Tex-related hub genes were mainly enriched in regulating leukocyte cell-cell adhesion and T-cell activation (**Figure 3A**). The top GO terms in the CC module were endocytic vesicle membrane, endocytic vesicle, and cytosolic small ribosomal subunit, and regarding MF, Tex-related hub genes were mainly enriched in MHC protein complex binding (**Figure 3A** **).** KEGG enrichment analysis containing T cell receptor signaling pathway, virus infection, and PD-L1 expression and PD-1 checkpoint pathway in cancer (**Figure 3B**). These results showed that the Tex-related gene list we found was efficient and reliable.

### 3.4 Transcription factor and small molecules for Tex reversion

**Figure 4:**
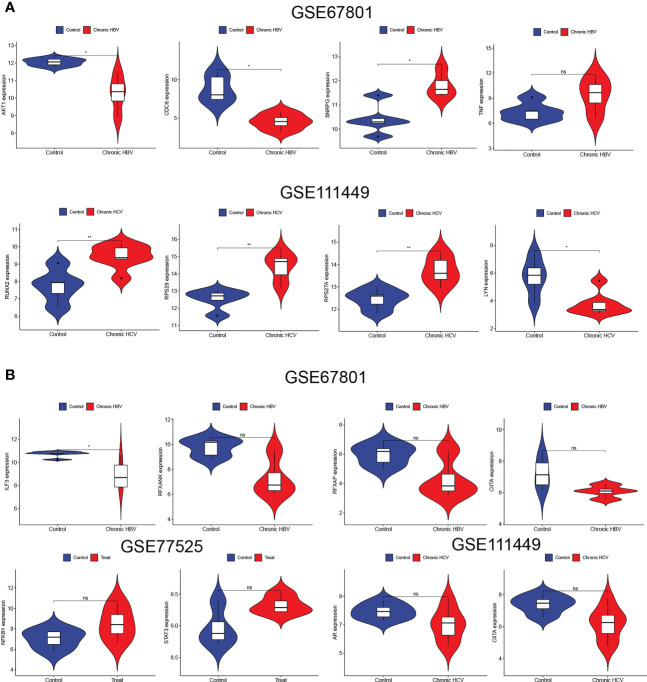
Validation of Tex-related transcription factors. **(A, B)** The Tex-related hub genes **(A)** and upstream transcription factors **(B)** predicted by the TRUST website were verified in the Tex GEO dataset GSE67801, GSE111449, and GSE77525.

To further explore the regulatory relationship of transcription factors upstream of Tex-related hub genes, which may contribute to T cell exhaustion, we used the TRUST website for further analysis. The results showed that the transcription factors such as ILF3, regulatory factor X-associated protein, STAT3, JUN, and RELA/NFKB1 regulated the hub genes’ expression, playing an important role in CD8+T exhaustion (**Table 2**). The hub genes and their predicted transcription factors were further validated in the data sets (**Figures 4A, B**). Targeting these transcription factors may help reverse T cells’ exhaustion state, restoring T cell function and increasing treatment effector for infections and tumors. We further identified potential small molecules reported to inhibit such TFs by the CLUE website (**Table 3**). In this section, we identified transcription factors critical for the induction of T cell exhaustion, providing potential targets for T exhaustion-reverse therapy.

**Table 2:** Transcription factors upstream of Tex-related hub genes.

Key TF	Description	P value	Q value	List of overlapped genes
ILF3	Interleukin enhancer binding factor 3, 90kDa	1.24E-05	0.000136	HLA-DRB1, HLA-DRA
RFXANK	Regulatory factor X-associated ankyrin-containing protein	6.16E-05	0.000226	HLA-DRA, HLA-DRB1
RFXAP	Regulatory factor X-associated protein	6.16E-05	0.000226	HLA-DRB1, HLA-DRA
RFX5	Regulatory factor X, 5 (influences HLA class II expression)	8.96E-05	0.000246	HLA-DRA, HLA-DRB1
CIITA	Class II, major histocompatibility complex, transactivator	0.000271	0.000595	HLA-DRB1, HLA-DRA
RELA	V-rel reticuloendotheliosis viral oncogene homolog A (avian)	0.00159	0.00254	TNF, PTPN6,AKT1
NFKB1	Nuclear factor of kappa light polypeptide gene enhancer in B-cells 1	0.00162	0.00254	PTPN6, AKT1, TNF
AR	Androgen receptor	0.00242	0.00333	CDC6, AKT1
E2F1	E2F transcription factor 1	0.00495	0.00605	TNF, CDC6
STAT3	Signal transducer and activator of transcription 3 (acute-phase response factor)	0.00554	0.00608	PTPN6, AKT1
JUN	Jun proto-oncogene	0.00608	0.00608	RUNX2, TNF

**Table 3:** Small molecules reported to inhibit Transcription factors in **Table 2**.

Iname	Idose	I time	Moa	Sample	Tas
BRD-A99218607	10 uM	24 h	NA	2	0.15
naproxol	10 uM	6 h	Anti-inflammatory	3	0.11
BRD-K63436783	10 uM	6 h	NA	3	0.07
BRD-K22384978	10 uM	6 h	NA	3	0.11
BRD-K04188046	10 uM	6 h	NA	5	0.23
Levothyroxine	2.22 uM	24 h	Thyroid hormone stimulant	2	0.21
Imatinib	10 uM	24 h	PDGFR inhibitor|Bcr-Abl inhibitor|KIT inhibitor	4	0.2
Ambrisentan	0.125 uM	24 h	Endothelin receptor antagonist	3	0.27
BRD-K28223745	4 uM	24 h	NA	3	0.13
VX-222	0.03 uM	24 h	HCV inhibitor	2	0.13
Alda-1	40 uM	6 h	Aldehyde dehydrogenase activator	2	0.12
Bucladesine	10 uM	6 h	NA	3	0.07
ICI-63197	10 uM	24 h	Phosphodiesterase inhibitor	2	0.05
Rutin	0.03 uM	24 h	Antioxidant|Capillary stabilizing agent|Nitric oxide scavenger	2	0.09
Tandutinib	10 uM	Six h	FLT3 inhibitor|KIT inhibitor|PDGFR inhibitor	5	0.33

NA: No annotation.

### 3.5 Construction and verification of the HCC prognostic model

**Figure 5:**
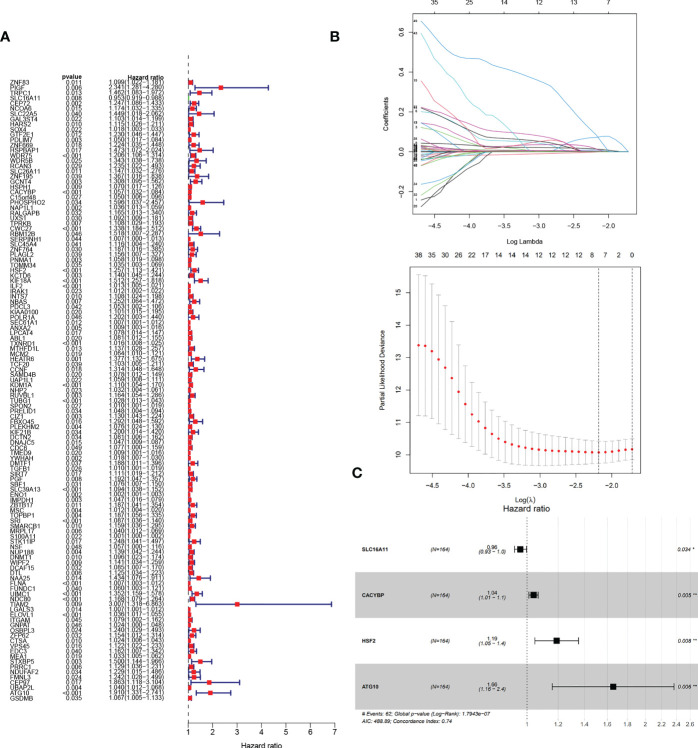
Construction of Tex-related HCC prognostic model. **(A)** The prognostic Tex-related genes extracted by univariate Cox regression analysis. **(B)** LASSO regression analysis and coefficients of Tex-related HCC prognostic model-constructed genes obtained by LASSO. **(C)** Multivariate analyses of model genes obtained from lasso regression analysis in the TCGA cohort. Hazard ratios are adjusted by the variables shown in this figure.

**Figure 6:**
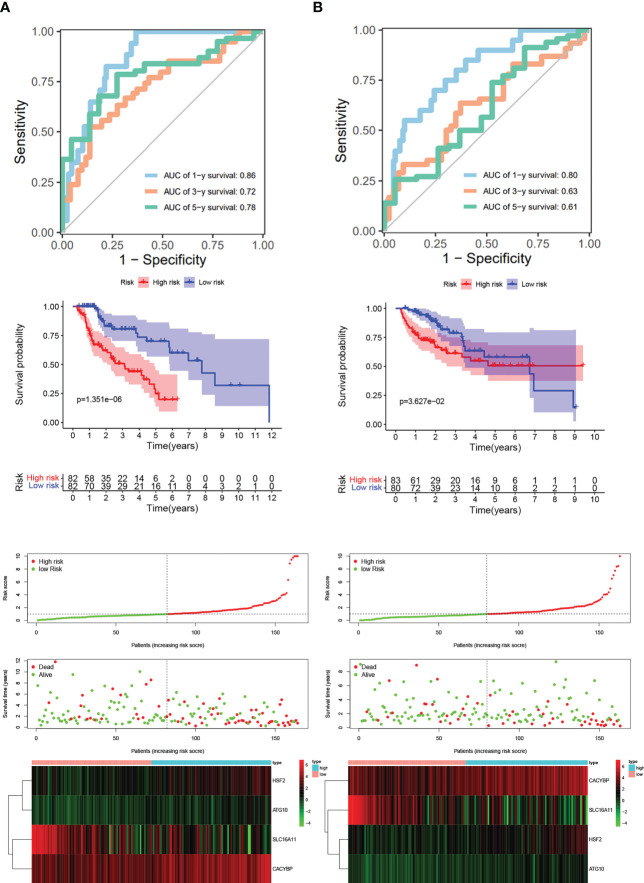
Evaluation and verification of Tex-related HCC prognostic model in TCGA training and testing sets. **(A)** Kaplan-Meier curve; ROC curves; Scatter plot of the risk score, overall survival, and corresponding heatmap in TCGA training set. **(B)** Kaplan-Meier curve; ROC curves; Scatter plot of the risk score, overall survival, and corresponding heatmap in TCGA testing set.

**Figure 7:**
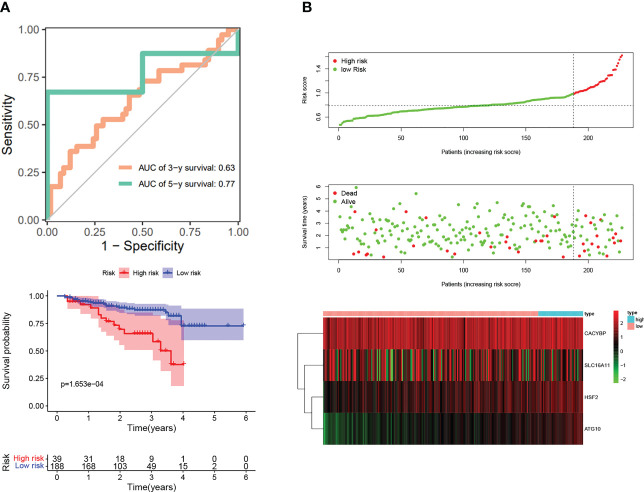
Validation of Tex-related HCC prognostic model in independent ICGC cohort. **(A)** Kaplan-Meier curve; ROC curves. **(B)** Scatter plot of the risk score, overall survival, and corresponding heatmap in ICGC verification set.

Next, we wanted to know whether Tex-related genes have predictive value for diagnosing and treating HCC. We collected 369 HCC samples and 50 cancer-adjacent control samples from the TCGA database. The DEGs in HCC were analyzed, and Tex-related DEGS were extracted for further analysis with | Log2Fold Change | > 1 and | adj. P value | < 0.05. Tex-related prognostic DEGs were identified by univariate cox regression (**Figure 5A**). Then least absolute shrinkage and selection operator (LASSO) was used to avoid overfitting (**Figure 5B**). Finally, four genes (SLC16A11, CACYBP, HSF2, and ATG10) were selected to construct the Tex-related prognostic model by multivariate Cox regression (**Figure 5C**, **Table 4**). We calculated risk score with the formula: risk score= SLC16A11 × (-0.0.3755) + CACYBP × (0.038092) + HSF2 × (0.173432) + ATG10 × (0.504545). In the TCGA cohort, low- and high-risk groups showed a significant difference in prognosis in the training and testing sets. The Kaplan-Meier curves showed that the OS of the high-risk group was significantly worse than that of the low-risk group. Time-dependent ROC curves were used to evaluate the sensitivity and specificity of the prognostic model by calculating the area under the ROC curve (AUC). In the training set, the 1-year, 3-year, and 5-year AUCs were 0.86, 0.72, and 0.78, and corresponding AUCs in the testing set were 0.80, 0.63, and 0.61 (**Figures 6A, B**). In addition, the independent data set based on ICGC further verified the accuracy and reliability of the model (**Figures 7A, B**). The clinical characteristics of the TCGA and ICGC databases are listed in **Table 5**. The above results indicated that the Tex-related prognostic model could accurately predict the prognosis of HCC. High-risk patients, based on risk scores, usually have a worse prognosis.

**Table 4:** Tex-related prognostic model by multivariate Cox regression.

Id	Coef	HR	HR.95L	HR.95H	P value
SLC16A11	-0.03755	0.963147	0.930365	0.997084	0.033566
CACYBP	0.038092	1.038827	1.011348	1.067052	0.005354
HSF2	0.173432	1.18938	1.046539	1.351717	0.007888
ATG10	0.504545	1.656231	1.158781	2.367231	0.005629

**Table 5:** The clinical characteristics of two cohort.

	TCGA cohort (n = 369)	LIRI-JP cohort (n = 231)
Age (median, range)	61 (16–90)	69 (31–89)
Gender (%)		
Female	121 (32.8%)	61 (26.4%)
Male	248 (67.2%)	170 (72.6%)
Grade (%)		
Grade 1	55 (14.9%)	NA
Grade 2	177 (48.0%)	NA
Grade 3	120 (32.5%)	NA
Grade 4	12 (3.3%)	NA
Unknown	5 (1.4%)	NA
Stage (%)		
I	170 (46.1%)	36 (15.6%)
II	85 (23.0%)	105 (45.5%)
III	85 (23.0%)	71 (30.7%)
IV	5 (1.4%)	19 (8.2%)
Unknown	24 (6.5%)	0 (0%)
Survival state		
OS days (median)	601	780

### 3.6 The risk signature and the clinicopathological features in HCC

**Figure 8:**
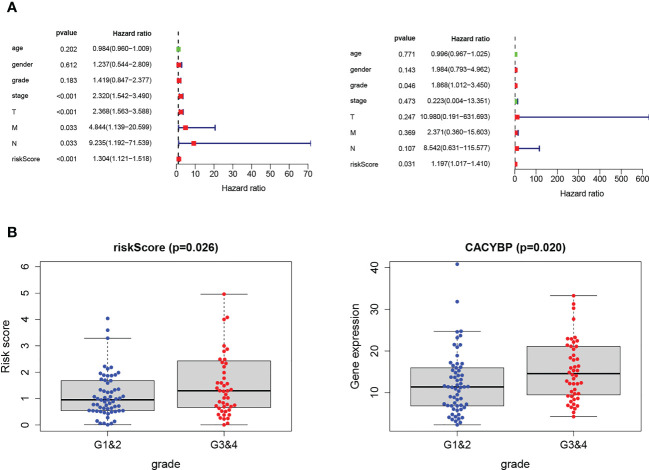
Validation of Tex-related HCC prognostic model in independent ICGC cohort. **(A)** Univariate and multivariate analyses indicated that the Grade and the risk score were independent prognostic factors of HCC patients. **(B)** The risk score and CACYBP had a significant correlation with Grade classification. *** indicates significant difference at p < 0.001.

We performed independent prognostic analyses to explore further the relationship between the risk model and clinical features, analyzing whether Tex signature can be used as an independent prognostic feature of HCC. In univariate Cox regression, the hazard ratio (HR) of the risk score was 1.301, and the 95% confidence interval (CI) was 1.121–1.518 (p < 0.001), while in multivariate Cox regression, they were 1.197 and 1.017-1.410 (p < 0.05). In addition, we identified that Grade was another independent prognostic parameter with HR 1.868 and 95%CI 1.012 - 3.450 (p < 0.05) (**Figure 8A**). Interestingly, it was observed that risk signatures could accurately reflect tumor grade characteristics and the expression level of CACYBP is also correlated with grade (p < 0.05) (**Figure 8B**). These results indicate that the HCC prognostic model constructed based on the four Tex-related genes can accurately reflect the degree of tumor progression, providing a basis for prognostic prediction and clinical decision-making.

### 3.7 The responsiveness of ICB immunotherapy

**Figure 9:**
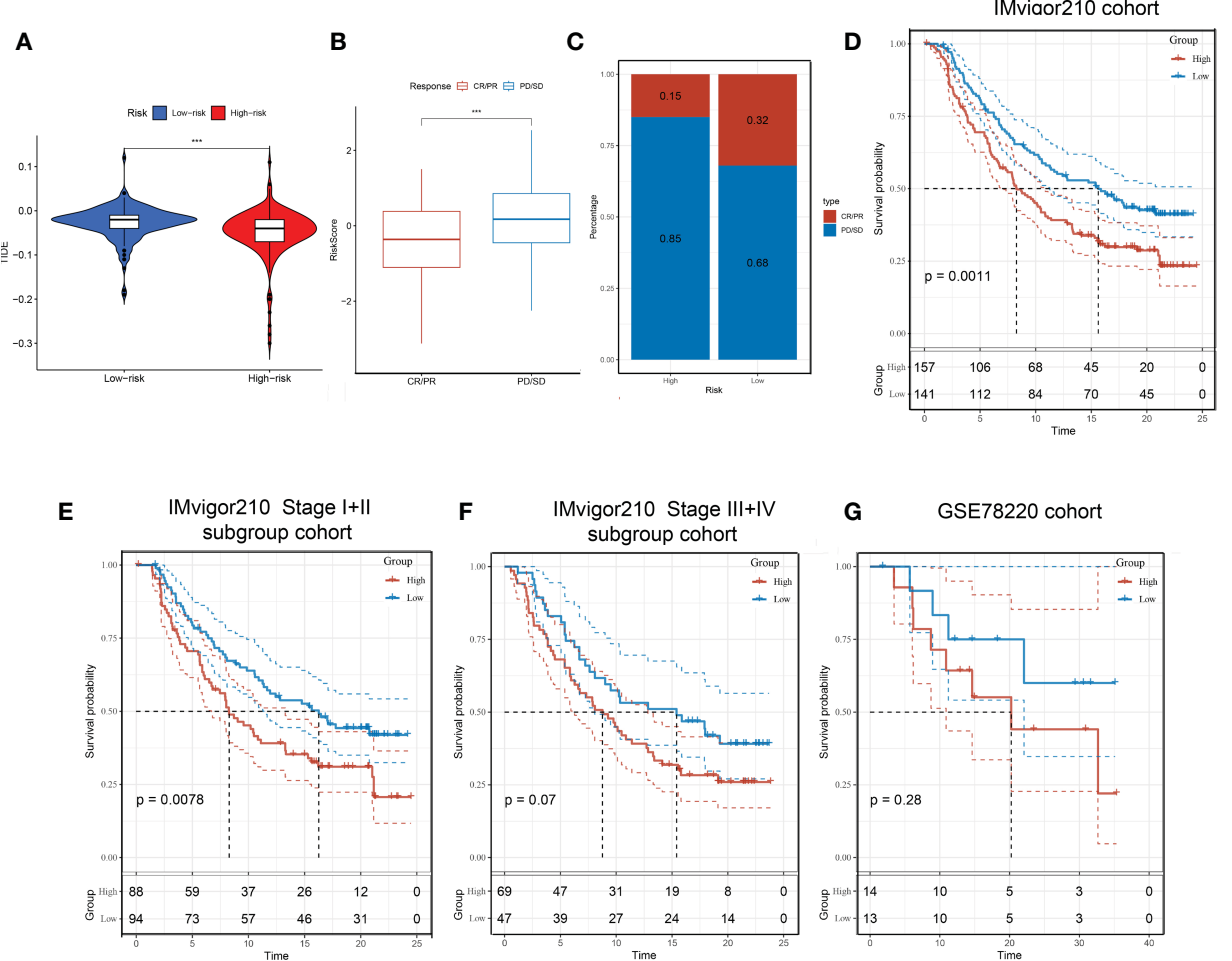
Immunotherapeutic responses in high- and low-risk groups with HCC. **(A)** Correlation between risk score and immunotherapy response. **(B)** In the IMvigor210 cohort, patients’ prognostic was related to Tex-related risk score. **(C)** Distribution of immunotherapy responses among high- and low-risk groups in the IMvigor210 cohort. **(D-F)** Prognostic differences among risk score groups in the IMvigor210 cohort **(D)**, Stage I+II subgroup **(E)**, and Stage III+IV subgroup **(F)**. **(G)** prognostic differences between high- and low-risk groups in the GSE78220 cohort.

We have established a Tex-related HCC prognostic model, and we wonder if this will help to guide clinical immunotherapy. Through the TIDE algorithm, we found that the high-risk group showed a promising response to immunotherapy (**Figure 9A**). Therefore, the Tex-related risk signature prognostic value was further tested based on IMvigor210 and GSE78220 cohorts ICB therapy. In the IMvigor210 cohort, patients’ respondence to anti-PD-L1 receptor blockers were classified into complete response (CR), partial response (PR), stable disease (SD), and progressive disease (PD) four outcomings. SD/PD patients acquired higher risk scores than CR/PR patients (**Figure 9B**). In the high-risk group, the percentage of SD/PD was higher than that in the low-risk group (**Figure 9C**). We observed that risk score demonstrated valuable prediction capacity and patients in the high-risk group showed significant clinical benefits than that of those in the low-risk group in the IMvigor210 cohort (p = 0.0011) (**Figure 9D**). Specifically, in the Stage I+II subgroup analysis, there were significant survival differences between the two risk groups (p = 0.0078) (**Figure 9E**). But not significantly different in Stage III+IV subgroup (p = 0.07) (**Figure 9F**). However, in the GSE78220 cohort, the patients between two groups didn’t demonstrate survival differences, which may because they were derived from melanomas data (**Figure 9G**). In conclusion, the results proofed that the Tex-related risk model makes sense in immunotherapy prediction.

### 3.8 Gene set enrichment analysis for Tex-related prognostic genes

**Figure 10:**
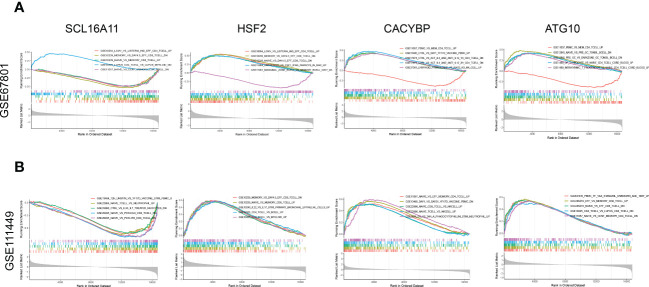
GSEA enrichment analysis for Tex-related HCC prognostic genes. **(A)** Enrichment analysis of immune-related data sets for SLC16A11, CACYBP, HSF2, and ATG10 based on the GSE67801 profile. **(B)** Enrichment analysis of immune-related data sets for SLC16A11, CACYBP, HSF2, and ATG10 based on the GSE111449 profile.

Four genes composing the prognostic model were analyzed based on GSE67801(**Figure 10A**) and GSE111449 (**Figure 10B**) databases to further investigate the role of Tex-related prognostic genes. Analysis was performed using immunologic signatures (GSEA C7) gene sets, and Top5 relevant data sets were displayed. The results showed that these four genes were strongly correlated with immune activation and CD8+T exhaustion, which proved the reliability of our model.

### 3.9 The CD8+T exhaustion verification by RT-qPCR and flow cytometry

**Figure 11:**
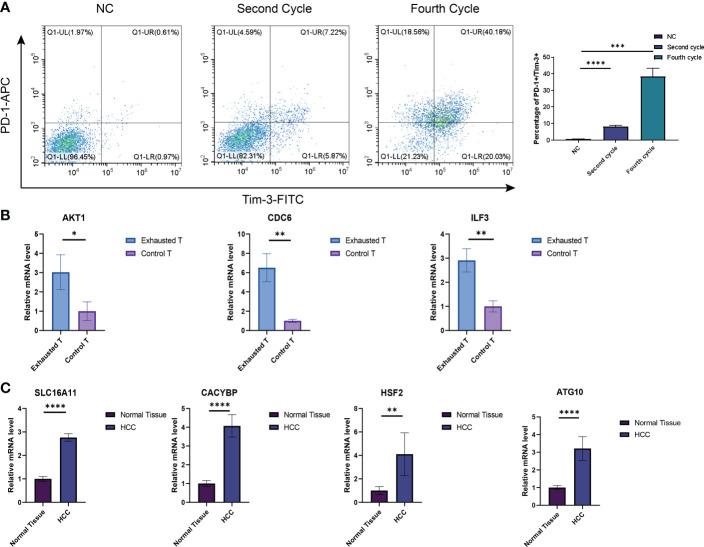
Verification of Tex signature by *in vitro* experiments. **(A)** Detection of the inhibitory receptor PD-1 and Tim-3 in T cells with different cycles of expansion. N=3 **(B)** qRT-PCR was used to detect exhaustion gene expression between the fourth cycle expanded T cells and NC cells. N = 3 **(C)** The expression of SLC16A11, CACYBP, HSF2, and ATG10 in HCC tissues and control tissues was detected by PCR. N = 5. *indicates significant difference at p < 0.05, ** indicates significant difference at p < 0.01, *** indicates significant difference at p < 0.001, **** indicates significant difference at P <0.0001.

The CD3^+^T cells were activated and amplified by CD3/CD28 magnetic beads to induce exhaustion. The T cell exhaustion state was compared between the fourth cycle stimulated T cells with those NC. The expressions of inhibitory receptors PD-1 and Tim-3 were detected by flow cytometry (**Figure 11A**), and qRT-PCR identified the expression of exhaustion-related hub genes and transcription factors (**Figure 11B**). Four Tex-related prognostic genes were compared between HCC tissues and normal tissues (**Figure 11C**). The results demonstrate the predictive value of Tex-related genes in the HCC prognosis model.

## 4 Discussion

With the popularization of immunotherapy and gene therapy, the prognosis of patients with HCC, especially those with advanced HCC, has been significantly improved. However, due to chronic viral infection, heterogeneity of tumors, and the special physiological structure of the liver, the immune microenvironment, the immune inhibition state of HCC, and the level of immune exhaustion have become important factors affecting the efficacy of immunotherapy (19). There is still a lack of effective biomarkers and prognostic models for HCC (20, 21). Therefore, a reliable and effective predictive model is critical for HCC treatment. This study established a prognostic model based on Tex-related genes and validated it in different databases. The results showed that the risk model could accurately reflect the prognosis of patients with HCC.

After acute infection and vaccination, naive T cells, activated by antigen, co-stimulation, and inflammation, proliferate exponentially to differentiate into effector T cells and memory T cells (22, 23). In patients with chronic infections and cancer, long-term exposure to persistent antigens and inflammation causes persistent stimulation of T cells, during which exhausted T cells gradually become unfunctional. Tex cells possess properties of progressive loss-of-effector function, poor memory recall, high and sustained inhibitory receptor expression, and metabolic and epigenetic reprogramming, distinguishing Tex from effector T cells and memory T cells (24–27). Studies on various tumor immune microenvironments, including liver cancer, melanoma, and lung cancer, have found that tumor-infiltrating T cells are usually exhausted, and the degree of exhaustion correlated with prognosis (28–30). Some key genes and transcription factors that induce Tex are gradually discovered. For example, NR4A and Tox family transcription factors were identified as key mediators of T cell exhaustion’s epigenetic and transcriptional programs (31, 32). The hypoxic microenvironment leads to cellular mitochondrial dysfunction, triggering the expression of transcriptional repressor BLIMP1 and producing Tex (33). Several approaches to partially reverse the T cell exhaustion phenotype through gene editing or small molecules and enhance the efficacy of immunotherapy have also been found. The classic example is immune checkpoint inhibitors help to suppress the expression of inhibitory receptors, reverse the exhausted T cell state, and restore tumor-infiltrating T cell (TIL) function (34). It has been proven to have a powerful anti-tumor effect. However, the mechanism of Tex induction is still not fully understood. The relationship between Tex and tumor and how to use Tex to guide tumor clinical treatment practice and predict patients’ prognosis still lacks effective models. In addition, like other immune cells, Tex T cells are heterogeneous, including progenitors and terminal subsets with unique characteristics. In conclusion, exploring the mechanism of Tex and constructing effective prognostic models may be of great significance for tumor therapy.

This study analyzed the Tex signature and its potential in HCC prediction. First, we constructed a Tex-related gene set based on GEO datasets and identified the hub genes through bioinformatic analysis. GO, KEGG, and GSEA enrichment analysis confirmed the promoting effect of these hub genes on Tex. Through prediction, we constructed transcription factor regulatory networks for hub genes. These transcription factors include: ILF3, RFXANK, RFXAP, RFX5, CIITA, RELA, NFKB1, AR, E2F1, STAT3 and JUN. Consistent with the conclusions of other studies, NFKB1/RelA heterodimers participated in NF-κB signaling pathway activation, which is an important factor for Tex production (35–37). Transcription factors CIITA, RFX5, RFXAP, and RFXANK mutations have been associated with Major histocompatibility complex (MHC) II deficiency, so they’re also associated with immune exhaustion. Tumor-derived cytokines such as IL-6 and IL-27 have promoted PD-L1+CD8+ T cells development through STAT1/STAT3 signaling (38, 39). ILF3 promotes the production of several tumors and is closely related to cellular senescence (40, 41). We also explored small molecules to target these transcription factors. Rutin, for example, a quercetin glycoside, possesses antioxidant and anti-aging properties. In conclusion, these results indicate that the Tex-related targets we have found are relatively accurate, and they may help to find ways for Tex state reversion and rejuvenate the immune system.

Further, Tex-related genes SLC16A11, CACYBP, HSF2, and ATG10 were selected to build a prognostic model for HCC, and excellent predictive capacity was demonstrated in the independent training, testing, and validation sets. SLC16A11, a protein in the endoplasmic reticulum and plasma membrane, is involved in pyruvate transport and lipid metabolism. Its abnormal expression or mutation is associated with various metabolic diseases such as type 2 diabetes, fatty liver, and obesity, all of which are risk factors for aging and cancer (42, 43). Therefore, the SLC16A11 gene may be an important target for regulating cellular senescence, immune cell exhaustion, and tumorigenesis. CACYBP is a calcyclin-binding protein. CACYBP has been reported as an independent prognostic factor in multiple cancers such as HCC and gastric cancer (44, 45). Moreover, it has also been identified as an immune-related biomarker with guiding value for the prognosis and immunotherapy effect in HCC, esophageal cancer, bladder cancer, and other tumors (45–47). A study based on single-cell sequencing has found that CACYBP can be used as an effective indicator to evaluate the efficacy of anti-PD-1 therapy and significantly up-regulated in exhausted CD8+T cells (48). HSF2 belongs to the HSF family of transcription factors. As a Heat shock protein, it has been reported to be associated with cellular stress and aging in diverse cells, including mesenchymal stem cells, skeletal muscle, and T cells (49–51). Through various bioinformatics, it has been related to immune cell infiltration and tumor immune microenvironment across different cancer types (52). Finally, the autophagy-related gene ATG10 regulated the formation of autophagosomes and participated in the anti-virus immune response (53). It has been identified as a downstream target of the PI3K/AKT/mTOR signaling pathway, is involved in various biological processes, and is closely related to aging (54). In conclusion, these Tex-related genes play an important role in immune exhaustion, cellular senescence, and tumorigenesis to provide a basis for the rationality of this prognostic model.

Although the study was based primarily on bioinformatics analysis, it has many implications. On the one hand, the study found the Tex gene set based on three etiologies-induced Tex cells data sets and explored its hub genes and transcription factors, which helps to reverse the characteristics of T cell exhaustion and to improve the effect of tumor immunotherapy. On the other hand, the prognostic model of HCC constructed by Tex-related genes has a good predictive function in both TCGA and ICGA data sets and can guide the clinical treatment of HCC. However, the study also has some limitations. First, we identified the Tex-related transcription factor regulating network and their targeting of small molecules, but the reversion effect lacked experimental verification. Second, Due to sequencing depth issues, some tumor-infiltrating T cells’ genes may be biased, and the role of some Tex genes may be neglected. Third, in HCC model construction and verification, separating CD8+T cells in HCC tissue is better for measuring the expression of hub genes or transcription factors. These shortcomings will be gradually overcome in the following research.

## 5 Conclusion

Our study demonstrated that Tex-related genes might provide accurate prediction for HCC patients in clinical decision-making, prognostic assessment, and immunotherapy. In addition, targeting the hub genes or transcription factors may help to reverse T cell function and enhance the effect of tumor immunotherapy.

## Data availability statement

The original contributions presented in the study are included in the article/**Supplementary Material**. Further inquiries can be directed to the corresponding author.

## Ethics statement

The samples acquired in this study and all of the experiments were approved by the Ethics Committee of Chinese PLA General Hospital (Beijing). The patients/participants provided their written informed consent to participate in this study.

## Author contributions

JS, GL, and LL conceptualized and designed the study. JS, GL, XY, YW, and MG performed the experiments and analyzed the data. XY, CL, and XG contributed significantly to the interpretation of the data. JS, GL, and LL wrote the manuscript. SL designed, supervised the research, and wrote the manuscript. All authors contributed to the article and approved the submitted version.
